# Damiana (*Turnera Aphrodisiaca*)

**Published:** 1879-02

**Authors:** 


Damiana (*Turnera Aphrodisiaca.*)—Dr. Summerlin, of Sunhill, Georgia, states that having seen this drug recommended for its aphrodisiac virtues he determined to give it a trial in the case of a patient, aged twenty-seven, who applied to him for treatment. The patient stated that a few years previously his right testicle became inflamed, compelling him to remain at home several days. After the swelling left, the testes became atrophied and tender to the touch. He had previously practiced onanism. The sexual desire had nearly left him. On examination the left testis was found to be soft and very small, the other normal. He was placed upon the usual treatment—nourishing food, nux vomica, iron and cantharides; but he did not appear to improve. He was then ordered to take one drachm of fluid extract of damiana three times a day. In a short time the testis began to enlarge and lose its sensitiveness. In the course of a month it had regained its normal size, and its functional activity was restored.—*Virginia Medical Monthly.*

